# Correction: Up-regulation of exosomal miR-125a in pneumoconiosis inhibits lung cancer development by suppressing expressions of EZH2 and hnRNPK

**DOI:** 10.1039/c8ra90078g

**Published:** 2018-10-10

**Authors:** Lin Zhang, Jiangfeng Li, Changfu Hao, Wei Guo, Di Wang, Jianhui Zhang, Youliang Zhao, Shuyin Duan, Wu Yao

**Affiliations:** Department of Occupational Hygiene, School of Public Health and Management, Healthy Shandong Collaborative Innovation Center for Major Social Risk Prediction and Governance, Weifang Medical University 7166 Baotong West Street Weifang 261024 China; Department of Occupational and Environmental Health, School of Public Health, Zhengzhou University 100 Science Avenue Zhengzhou 450001 China yaowu@zzu.edu.cn +86-371-67781922; School of Basic Medicine, Zhengzhou University 100 Science Avenue Zhengzhou 450001 China; Department of Occupational Disease, Henan Provincial Institute of Occupational Health Middle Street of Kangfu Zhengzhou 450052 China

## Abstract

Correction for ‘Up-regulation of exosomal miR-125a in pneumoconiosis inhibits lung cancer development by suppressing expressions of EZH2 and hnRNPK’ by Lin Zhang *et al.*, *RSC Adv.*, 2018, **8**, 26538–26548.

The authors regret that the affiliations of the authors were wrongly designated in the original manuscript. The corrected list of affiliations is as shown below.

The Royal Society of Chemistry apologises for these errors and any consequent inconvenience to authors and readers.

## Supplementary Material

